# Avibactam–Cyclodextrin Inclusion Complexes: Computational and Thermodynamic Insights for Drug Delivery, Detection, and Environmental Scavenging

**DOI:** 10.3390/molecules30163401

**Published:** 2025-08-18

**Authors:** Jackson J. Alcázar, Paola R. Campodónico, René López

**Affiliations:** 1 Centro de Química Médica, Facultad de Medicina Clínica Alemana, Universidad del Desarrollo, Santiago 7780272, Chile; pcampodonico@udd.cl; 2 Departamento de Paciente Crítico, Clínica Alemana de Santiago, Santiago 7650568, Chile; rene.lopez@udd.cl; 3 Grupo Intensivo, Instituto de Ciencias e Innovación en Medicina, Facultad de Medicina Clínica Alemana, Universidad del Desarrollo, Santiago 7780272, Chile

**Keywords:** avibactam, β-Lactamase inhibitor, supramolecular derivatization, cyclodextrin inclusion complex, molecular dynamics simulation, isothermal titration calorimetry

## Abstract

The escalating crisis of multidrug resistance, together with the persistence of antibiotic residues in clinical and environmental matrices, demands integrated strategies that couple sensitive detection, efficient decontamination, and controlled delivery. However, current techniques for quantifying avibactam (AVI)—a broad-spectrum β-lactamase inhibitor—such as HPLC-UV lack the sensitivity and specificity required for both therapeutic drug monitoring and environmental surveillance. Encapsulation of AVI within cyclodextrins (CDs) may simultaneously enhance its stability, bioavailability, and detectability, while the high binding affinities of CDs position them as molecular traps capable of scavenging residual AVI. In this study, the inclusion complexation of AVI with various CDs was examined through molecular dynamics (MD) simulations, experimental isothermal titration calorimetry (ITC), and non-covalent interaction (NCI) analysis. Stable 1:1 inclusion complexes were observed between AVI and β-cyclodextrin (β-CD), 2,6-dimethyl-β-cyclodextrin (DM-β-CD), and 2-hydroxypropyl-β-cyclodextrin (HP-β-CD), with standard Gibbs free energies of binding (ΔG°) of –3.64, –3.24, and –3.11 kcal/mol, respectively. In contrast, γ-cyclodextrin (γ-CD) exhibited significantly weaker binding (ΔG° = –2.25 kcal/mol). DFT-based NCI analysis revealed that cooperative interaction topology and cavity complementarity, rather than the sheer number of localized contacts, govern complex stability. Combined computational and experimental data establish β-CD derivatives as effective supramolecular hosts for AVI, despite an entropic penalty in the DM-β-CD/AVI complex. These CD–AVI affinities support the development of improved analytical methodologies and pharmaceutical formulations, and they also open avenues for decontamination strategies based on molecular trapping of AVI.

## 1. Introduction

Multidrug-resistant Gram-negative bacilli (MDR-GNB) represent a major threat in intensive care units (ICUs) due to their resistance to a broad spectrum of antibiotics, including carbapenems [1]. In particular, infections caused by carbapenem-resistant Enterobacterales have been associated with a two-fold increase in mortality [2]. The global escalation of bacterial resistance, both reported and projected [3], is compounded by the persistence of antibiotic residues in hospital effluents and natural waterways, which sustains selective pressure for further resistance [4]. Together, these factors highlight the pressing need for the development of advanced antibiotic delivery systems and effective strategies to remove antibiotic residues from aquatic environments [5,6].

Among the promising therapeutic options, avibactam (AVI) has gained attention for its broad-spectrum activity against Ambler class A, C, and certain class D β-lactamases [7,8], alongside synergistic effects when combined with other antibiotics [9,10]. Notably, AVI is a core component of Zavicefta^®^, a last-line therapy composed of ceftazidime (CAZ) and AVI, in which AVI restores CAZ’s efficacy against carbapenemase-producing organisms [11,12,13].

**Scheme 1:**
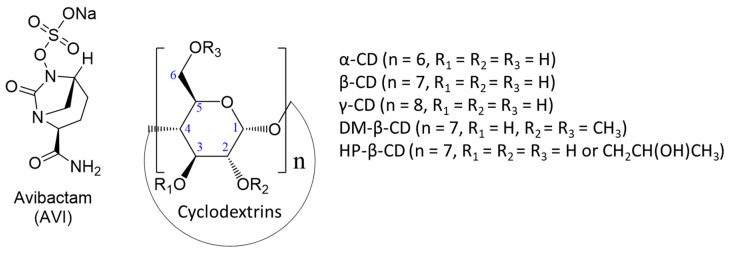
Representative structures of avibactam (AVI) and cyclodextrins: α-CD, β-CD, γ-CD, Heptakis(2,6-di-O-methyl)-β-CD (DM-β-CD), and 2-Hydroxypropyl-β-CD (HP-β-CD).

Structurally, AVI is a non-β-lactam β-lactamase inhibitor based on a diazabicyclo[3.2.1]octane scaffold—specifically, [(2S,5R)-2-carbamoyl-7-oxo-1,6-diazabicyclo[3.2.1]octan-6-yl] hydrogen sulfate (see Scheme 1) [14]—offering enhanced rigidity and hydrogen-bonding capabilities [15]. It operates through a slowly reversible acylation mechanism, allowing for the regeneration of intact AVI [16,17]. However, despite its pharmacological promise, its modest aqueous solubility and stability pose challenges for dosing in critically ill patients, where precise pharmacokinetic (PK) and pharmacodynamic (PD) control is essential [18].

To address these clinical challenges, therapeutic drug monitoring (TDM) has been proposed as a means to optimize dosing and reduce the risk of resistance emergence among Gram-negative pathogens [19]. TDM relies on robust bioanalytical methods capable of accurately quantifying plasma drug levels, particularly for antibiotics with narrow therapeutic windows [20].

Currently, AVI quantification is primarily achieved through liquid chromatography–tandem mass spectrometry (LC–MS/MS), a technique known for its high sensitivity and specificity [21,22,23,24]. However, the high cost and limited accessibility of MS-based platforms hinder their widespread implementation. In contrast, high-performance liquid chromatography with ultraviolet detection (HPLC-UV) represents a more accessible alternative [25], though often at the expense of sensitivity and selectivity—especially when analyzing complex biological matrices such as plasma. Hence, there is a pressing need for alternative analytical strategies that maintain high performance without reliance on MS instrumentation.

In this context, cyclodextrins (CDs) emerge as promising tools. These cyclic oligosaccharides (see Scheme 1) can form inclusion complexes with various drugs, thereby modifying their physicochemical properties and enhancing analytical detectability [26,27,28,29,30]. The incorporation of CDs into analytical platforms has improved the quantification of several antibiotics by increasing solubility, stability, and signal response.

Beyond analytical applications, CDs are also widely employed as excipients in oral and parenteral drug formulations [31,32,33]. Their ability to encapsulate guest molecules enhances aqueous solubility, protects labile functional groups, and facilitates controlled or sustained release. Moreover, CDs can be leveraged in environmental remediation: soluble or immobilized forms of CDs have demonstrated removal efficiencies of up to 90% for ß-lactam antibiotics in wastewater, thereby reducing environmental selective pressure [34,35]. These dual roles—in pharmaceutical enhancement and antibiotic scavenging—highlight the importance of understanding AVI–CD interactions.

CDs are cyclic oligosaccharides of 6 (α-CD), 7 (β-CD), or 8 (γ-CD) D-glucose units linked by α-1,4 glycosidic bonds (see Scheme 1), forming a toroidal structure with a relatively hydrophobic cavity and hydrophilic exterior [36,37,38]. This unique architecture allows for the inclusion of hydrophobic guest molecules, thus enhancing solubility and stability, and modulating PK profiles—particularly in their methylated (DM-β-CD) or hydroxypropylated (HP-β-CD) derivatives [39,40,41,42,43].

In this study, we explore the formation of inclusion complexes between avibactam and various CDs (α-, β-, γ-, 2,6-dimethyl-β-, and 2-hydroxypropyl-β-CD) using a multidisciplinary approach that integrates molecular dynamics (MD) simulations, isothermal titration calorimetry (ITC), and density functional theory (DFT). Elucidating the thermodynamic signatures and binding topologies of these host–guest systems lays the foundation for future CD-based analytical platforms, drug delivery systems, and environmental decontamination strategies.

## 2. Results and Discussion

### 2.1. Molecular Dynamic

**Figure 1:**
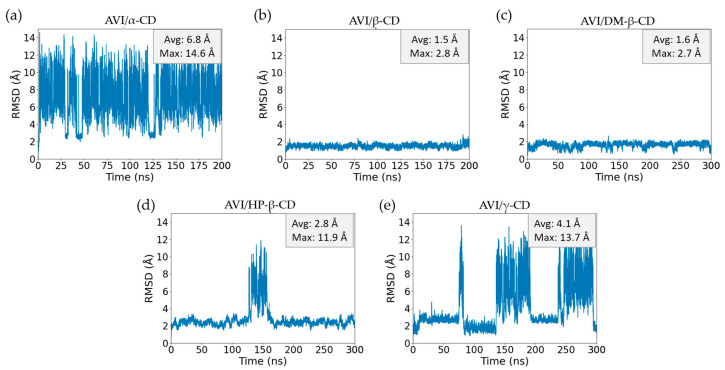
Root mean square deviation (RMSD, Å) over time (ns) for avibactam (AVI) with cyclodextrins: (**a**) α-CD, (**b**) β-CD, (**c**) DM-β-CD, (**d**) HP-β-CD, and (**e**) γ-CD. Avg represents the average RMSD in Å, and Max represents the maximum RMSD in Å, as indicated in each graph.

**Figure 2:**
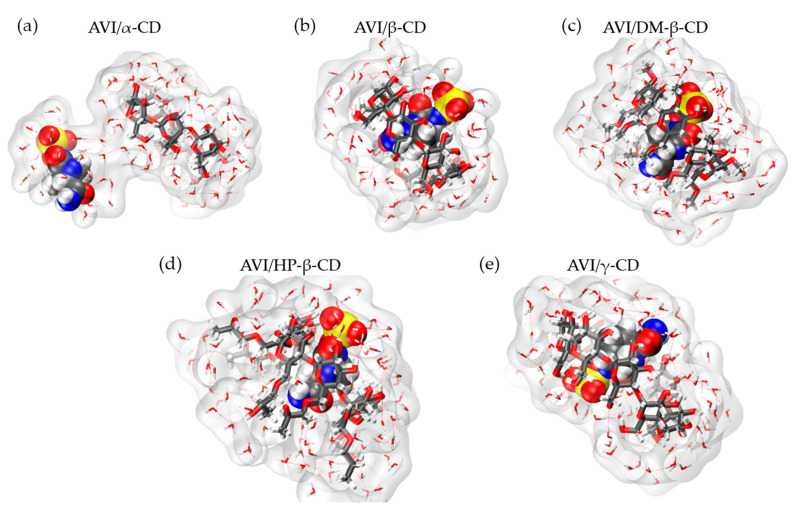
Representative snapshots of the last frame from the molecular dynamics simulations for avibactam (AVI) complexed with different cyclodextrins (CDs): (**a**) α-CD, (**b**) β-CD, (**c**) DM-β-CD, (**d**) HP-β-CD, and (**e**) γ-CD. The final configurations correspond to 200 ns for (**a**,**b**), and 300 ns for (**c**–**e**). AVI is depicted using the van der Waals (VDW) representation, with standard atomic colors: carbon (gray), oxygen (red), nitrogen (blue), and sulfur (yellow). The CDs are represented in licorice format. The simulations were performed in an explicit water environment. Three-dimensional structures can be visualized at http://doi.org/10.6084/m9.figshare.28773743 (accessed on 30 July 2025).

Figure 1 presents the root mean square deviation (RMSD) over time for AVI in the presence of different CDs, while Figure 2 provides snapshots of the final frames (200 ns for α- and β-CD, and 300 ns for the others) from each MD simulation. In Figure 2, AVI is represented using a van der Waals (VDW) model with standard atomic colors—carbon (gray), oxygen (red), nitrogen (blue), and sulfur (yellow)—whereas the CDs are displayed in licorice format. Together, these figures offer both quantitative (RMSD) and qualitative (final structural conformations) insights into each AVI–CD complex.

The AVI/α-CD system (Figure 1a) exhibits the highest RMSD fluctuations, averaging 6.8 Å with a maximum of 14.6 Å, indicative of weak and transient binding. The final snapshot in Figure 2a reinforces this notion by showing AVI only loosely associated with the relatively small α-CD cavity. In contrast, AVI/β-CD (Figure 1b) presents markedly lower RMSD values (average of 1.5 Å, maximum of 2.8 Å), consistent with a stable binding mode evident in Figure 2b. Similar stability is observed for AVI/DM-β-CD (Figure 1c), which shows low RMSD values (average of 1.6 Å, maximum of 2.7 Å). Notably, although the bisulfate group of AVI in both β-CD (Figure 2b) and HP-β-CD (Figure 2d) points toward the secondary face, it remains outside the cavity. In contrast, in DM-β-CD (Figure 2c), this group is fully included, interacting directly with the methylated substituents at the secondary rim. This distinction highlights the importance of functionalizing the hydroxyl groups of β-CD for effective guest encapsulation, particularly at the C2 position (see Scheme 1). When the C2 hydroxyl is unmodified, as seen in Figure 2b,d, the bisulfate group remains external, whereas the presence of a 2-methoxy group results in the bisulfate group being more deeply embedded.

The AVI/HP-β-CD system (Figure 1d) displays a clear temporary dissociation event around 150 ns, confirmed by monitoring the simulation trajectory, where the RMSD peaks at 11.9 Å. After 170 ns, the complex re-forms, demonstrating that once AVI reorients, stable binding is restored. Lastly, the AVI/γ-CD system (Figure 1e) exhibits moderate instability, with three intervals (75–85 ns, 140–190 ns and 240–290 ns) of elevated RMSD indicating repeated dissociation–reassociation episodes accompanied by conformational rearrangements. The final snapshot in Figure 2e reveals a distinct AVI–CD interaction conformation compared to those observed in the other cyclodextrin complexes. This difference may arise because the relatively large γ-CD cavity allows AVI to better interact with its bisulfate group and the narrower primary face of γ-CD. However, this conformation could be transient rather than final and may exchange with others, given the highly dynamic nature of the system indicated by its elevated RMSD values.

Overall, these MD simulations underscore the pivotal role of cyclodextrin cavity size and functionalization at the secondary rim in establishing and maintaining stable host–guest complexes with AVI. The dynamic behaviors observed, which range from transient, loose binding (α-CD) to more stable encapsulations (β-CD, DM-β-CD, HP-β-CD, and γ-CD), highlight how even subtle structural differences at the C2 position could significantly influence the orientation of the guest and overall stability within the cavity. In the following section, we discuss the ITC results, which quantify the binding interactions under real conditions and provide additional insight into the thermodynamic underpinnings of these AVI-CD complexes.

### 2.2. Isothermal Titration Calorimetry

**Figure 3:**
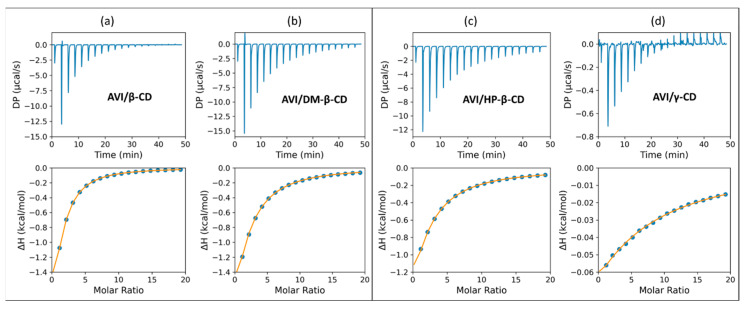
Experimental isothermal titration calorimetry (ITC) of avibactam (AVI) with cyclodextrins (β-CD, DM-β-CD, HP-β-CD, and γ-CD): Graphs (**a**–**d**) show the differential power (DP) over time (top panels) and the integrated, normalized heat plots (bottom panels) at AVI concentrations corresponding to molar ratios ranging from 1 to 19. These experiments were conducted with a fixed cyclodextrin concentration of 1 × 10^−3^ M. Baseline corrections and contributions from non-specific AVI–water and CD–water interactions were subtracted to isolate the specific heat signals from the AVI–cyclodextrin interactions. The observed heats of interaction align with a 1:1 binding model, as indicated by the orange fitting curves in the normalized heat plots. All titrations were performed at 298.15 K in water.

In order to experimentally quantify the AVI-CD binding interactions, the ITC method was employed under two different concentration regimes, as illustrated in Figure 3. Distinct calorimetric profiles were obtained for each CD, and the resulting differential power curves, along with the integrated, normalized heat plots, reveal the nature and strength of AVI–CD complexation.

As shown in Figure 3, the heat signals are consistent with a 1:1 binding model across all systems, as indicated by the fitted curves. Differences in the shape and intensity of the curves suggest that AVI exhibits varying affinities depending on the structural features of the CDs.

The thermodynamic parameters summarized in Table 1 were obtained from the non-linear fitting of the integrated heat data shown in Figure 3. These values quantitatively support the qualitative trends observed in the calorimetric profiles. The equilibrium binding constants (*K*_1:1_) indicate that β-CD exhibits the strongest affinity for AVI (*K*_1:1_ ≈ 467), followed by DM-β-CD (*K*_1:1_ ≈ 236) and HP-β-CD (*K*_1:1_ ≈ 192), whereas γ-CD demonstrates a markedly weaker interaction (*K*_1:1_ ≈ 44). These trends are mirrored in the free energy changes, where more negative values correspond to more spontaneous processes; in particular, β-CD features the most negative ΔG° (–3.64 kcal/mol), underscoring its robust binding to AVI. Notably, these affinities fall within the same order of magnitude as those reported for other diazabicyclo-based β-lactamase inhibitors (DBOs) and for model naphthalenesulfonate probes (ANS) [44,45,46], underscoring the consistency of CD host behavior toward structurally related guests.

A decomposition of the binding thermodynamics highlights the impact of secondary-rim alkylation. Methylated and hydroxypropylated β-CDs exhibit significantly more exothermic enthalpies (ΔH° = –7.65 and –7.08 kcal mol^−1^, respectively) than native β-CD (–4.57 kcal mol^−1^), reflecting additional dispersion forces, hydrophobic desolvation, and newly accessible host–guest hydrogen bonds afforded by C2/C6 substitution. The concomitant increase in the entropic penalty (larger –TΔS°, Table 1) indicates greater conformational confinement of the macrocycle–guest ensemble and substantial solvent reorganization, illustrating classical enthalpy–entropy compensation. This stability enhancement is consistent with the mechanistic picture proposed in a computational study [38], which ascribed the higher affinities of methyl-β-CD complexes to a net gain in intermolecular hydrogen bonding that outweighs disruption of the native intra-annular H-bond network and any host dimerization.

γ-CD, in contrast, shows the lowest binding constant, derives only a modest enthalpic benefit (–1.42 kcal/mol), and experiences a slight unfavorable entropic change (–0.83 kcal/mol), culminating in the weakest net binding. The weaker interaction observed for γ-CD may be attributed to steric mismatch or lower cavity complementarity with the guest.

These thermodynamic observations align with the MD simulation findings. The MD data reveal that β-CD and DM-β-CD form stable complexes with AVI, as evidenced by consistently low RMSD values and well-defined binding modes in the final simulation snapshots. In contrast, HP-β-CD exhibits transient dissociation events—observed as temporary increases in RMSD—while γ-CD undergoes repeated dissociation–reassociation episodes, reflecting its overall weaker interaction. The dynamic behaviors captured by the MD simulations thus complement the ITC data, collectively highlighting β-CD and DM-β-CD as the most promising candidates for forming stable AVI–CD complexes.

### 2.3. Non-Covalent Interactions

**Figure 4:**
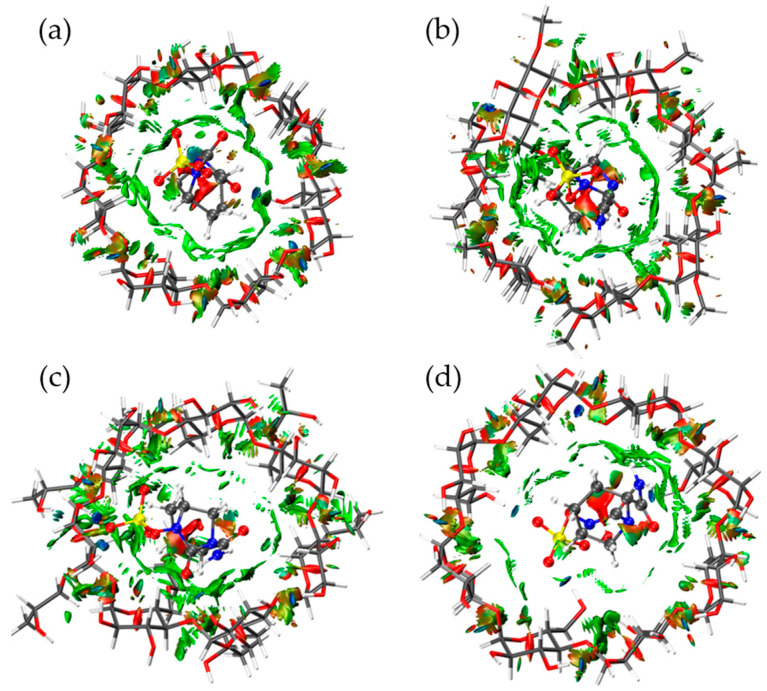
Color-filled RDG isosurface for intermolecular host–guest interactions between (**a**) AVI/β-CD, (**b**) AVI/DM-β-CD, (**c**) AVI/HP-β-CD, and (**d**) AVI/γ-CD. The different types of interaction regions are highlighted in different colors; blue: attractive interactions like hydrogen bonds, green: slightly attractive vdW interactions, and red: repulsive steric interactions. Three-dimensional visualizations of the structures are available at http://doi.org/10.6084/m9.figshare.28773743 (accessed on 30 July 2025).

The non-covalent interaction (NCI) analysis was performed on the last frame of the molecular dynamic simulation, optimized using DFT with the low-cost B97-3C method. Figure 4 shows strong attractive interactions are depicted in blue, slightly attractive vdW interactions in green, and steric repulsive interactions in red. For the optimized final frames, differences were observed in the vdW interaction surfaces (green regions). For instance, Figure 4b, which corresponds to the AVI/DM-β-CD complex, shows a larger area and a higher number of attractive vdW interactions compared to the other complexes (Figure 4a,c,d). Notably, the AVI/γ-CD complex (Figure 4d) exhibited the smallest surface of attractive vdW interactions. This observation is consistent with the enthalpy values obtained from ITC (Table 1), where AVI/DM-β-CD showed the most favorable ΔH° (–7.65 kcal/mol), while AVI/γ-CD had the least favorable ΔH° (–1.42 kcal/mol).

In terms of strong attractive interactions, all complexes tend to establish at least one strong interaction, such as ion–dipole or hydrogen bonding. Interestingly, despite exhibiting the least favorable ΔH° and the lowest vdW interaction surface, the AVI/γ-CD complex showed the highest number of strong interactions compared to the others (Figure 4). This may be attributed to the fact that these strong interactions in the AVI/γ-CD complex are primarily located at the rim of the host cavity, leaving a significant portion of the internal cavity unoccupied and lacking effective contact with the guest. This spatial mismatch likely results in weaker anchoring and suboptimal host–guest recognition. Consequently, the guest retains a high degree of translational freedom within the cavity, potentially increasing its kinetic energy relative to the potential energy contribution from non-covalent stabilization. In agreement with this interpretation, the AVI/γ-CD complex also displayed the highest frequency of dissociation events during molecular dynamics simulations (Figure 2e), highlighting that a greater number of localized strong interactions does not necessarily correlate with enhanced overall complex stability.

In summary, the NCI analysis provided valuable insights into the nature and distribution of non-covalent interactions within each host–guest complex. While strong interactions were present in all systems, their spatial arrangement and the extent of vdW contact surfaces played a more decisive role in determining complex stability. The AVI/DM-β-CD complex, with its broader vdW interaction surface and more favorable enthalpic profile, demonstrated enhanced host–guest complementarity and greater stability. In contrast, the AVI/γ-CD complex, despite having more strong localized interactions, lacked sustained contact throughout the cavity, leading to increased guest mobility and a higher propensity for dissociation. These findings emphasize the importance of cooperative and spatially distributed interactions—rather than isolated strong contacts—in achieving thermodynamically stable host–guest systems.

## 3. Materials and Methods

### 3.1. Molecular Dynamics (MD) Calculation

To prepare the molecular systems, the structures of AVI and CDs (α-CD, β-CD, DM-β-CD, HP-β-CD, and γ-CD) were drawn and optimized using the MMFF94 force field with the Avogadro 1.2.0 software [47]. Subsequently, the MD simulations of each AVI and cyclodextrin system (AVI/α-CD, AVI/β-CD, AVI/DM-β-CD, AVI/HP-β-CD, and AVI/γ-CD) were performed using NAMD 3 [48]. The topology and parameter files for the host molecules were obtained using the Ligand Reader & Modeler module from CHARMM-GUI [49,50]. AVI required parameterization, which was carried out using the Force Field Toolkit (FFTK) plugin [51] of VMD (Visual Molecular Dynamics) version 1.9.4 [52]. The MP2 6-31G* [53,54,55] method was employed for accurate force field parameter derivation, and these quantum mechanical calculations were performed using ORCA version 4.1.1 [56].

Molecular dynamics were carried out starting from a host–guest associated structure, which was initially docked using the AutoDock program (Version 4.2) [57]. The docking simulations were performed using the Lamarckian genetic algorithm (LGA), optimizing the conformational exploration of the guest while keeping the receptor fixed. A computational grid of 60 × 60 × 60 points was defined to ensure a uniform search area. To enhance search efficiency, a population size of 1500 was established for each run, with a total of 1000 runs, while other parameters were kept at default values. For these calculations, Merz–Kollman (MK) charges [58] were employed, derived from the previously optimized structures of the individual monomers. The calculation of these charges was performed using the Multiwfn program (Version 3.8) [59], following the approach described by Niklas et al. [38].

Following the docking calculations, the system underwent molecular dynamics equilibration at 298.15 K in the NPT ensemble (constant number of particles, pressure, and temperature) containing 2901 water molecules. Basic dynamics settings included a 1 fs timestep, with non-bonded interactions calculated every 2 steps and full electrostatics every 4 steps. Langevin dynamics were employed for temperature control, using a damping coefficient of 1 ps^−1^ and a bath temperature of 298.15 K. Pressure control was managed using a Langevin piston set to 1.01325 bar, with a period of 200 fs and a decay time of 100 fs.

Periodic boundary conditions were applied with cell vectors set to 34 Å in each dimension, and Particle Mesh Ewald (PME) method was used for long-range electrostatics, with a tolerance of 10^−6^ and grid spacing of 1.0 Å. The system was minimized for 15,000 steps to resolve any steric clashes, followed by velocity reinitialization to the target temperature.

To ensure proper equilibration and sampling, the production runs were conducted for 200 ns for AVI/α-CD and AVI/β-CD, while a longer simulation time of 300 ns was employed for AVI/DM-β-CD, AVI/HP-β-CD, and AVI/γ-CD due to their higher structural flexibility.

All input files related to docking, parameterization, and molecular dynamics simulations—including topology, parameter, and configuration files—are provided in the Supplementary Material.

### 3.2. Isothermal Titration Calorimetry (ITC) Analysis

ITC experiments were conducted in duplicate using a Malvern Panalytical PEAQ-ITC system to characterize the binding interactions between AVI and CDs (β-CD, DM-β-CD, HP-β-CD, and γ-CD). All titrations were performed at 298.15 K (25 °C) in water. The instrument was calibrated according to the guidelines of manufacturer prior to use, and the reference cell was filled with deionized water.

AVI was obtained from Pfizer with a purity of 99.6% in its sodium salt form. CDs were purchased from AK Scientific with the following purity levels and degree of substitution (DS): β-CD (98%), DM-β-CD (98%, DS ≈ 14), HP-β-CD (98%, DS ≈ 7, random), and γ-CD (99%). Stock solutions of the CDs were prepared at concentrations of 1 × 10^−2^ M for the first set of titrations, where AVI concentrations ranged from molar ratios of 0.1 to 1.9, and 1 × 10^−3^ M for the second set, covering molar ratios from 1 to 20. The AVI stock solution was prepared in deionized water, and all samples were filtered before loading into the instrument to prevent clogging of the injection syringe.

The titration was performed with an initial delay of 60 s to ensure baseline stability, followed by 19 injections. The first injection was 0.4 µL, administered over 0.8 s, while the subsequent 18 injections consisted of 2.0 µL each, with an injection duration of 4.0 s and an interval of 150 s between injections. The stirring speed was set to 750 rpm, and the reference power was maintained at 10 µcal/s in high feedback mode to optimize sensitivity and minimize noise. The filter period was set to 5 s to ensure proper signal processing.

The differential power (DP) signal was recorded throughout the titration, and heat data were integrated after baseline correction. Non-specific heat contributions from AVI–water and CD–water interactions were subtracted to isolate the specific heat signatures associated with AVI-CD binding. The integrated heat values were plotted against the molar ratio and fitted to a 1:1 binding model. This analysis provided key thermodynamic parameters, including the binding constant (*K*), enthalpy change (ΔH), entropy change (ΔS), and stoichiometry (N). The observed binding profiles allowed for a comparative assessment of the interaction strengths between AVI and the different CDs.

### 3.3. Non-Covalent Interaction (NCI) Analysis by DFT Method

The electronic structure of the host–guest complexes was optimized using the low-cost B97-3C method [60] within the ORCA program package (program version 6.0.0) [61,62]. All optimizations were performed under implicit water solvent conditions, modeled using the conductor-like polarizable continuum model (CPCM) with the COSMO epsilon function [63], following the TightSCF convergence protocol. To ensure that each electronic structure corresponded to a local energy minimum, vibrational frequency analyses were conducted and confirmed by the presence of only positive vibrational modes.

The choice of the B97-3C method was based on its recognized balance between computational efficiency and reliability [60], which renders it particularly suitable for evaluating host–guest systems [38,64,65].

Subsequently, the non-covalent interactions within these systems were examined using the reduced density gradient (RDG) method, following the protocol established by Yang et al. [66] and implemented in the Multiwfn program [59]. In this analysis, the electron density (ρ) of the complexes was mapped using the following equation:

(1) $$\mathrm{RDG}\;\left(r\right)=\;\frac{1}{{2(3\pi^{2})}^{1/3}}\frac{\left|\nabla \rho (r)\right|}{{\rho (r)}^{4/3}}$$

This formulation takes into account the sign of the second largest eigenvalue of the electron density’s Hessian matrix (denoted as sign (λ_2_)) multiplied by ρ, thereby distinguishing between various non-covalent interactions at the critical bond regions identified through Bader’s atoms in molecules (AIM) theory [67]. An isovalue of 0.6 was chosen consistently, as it was found to be sufficient for constructing the RDG isosurface that effectively highlights all weak interactions in three-dimensional space.

The optimized structures and input files for the DFT and NCI analyses are also included in the Supplementary Material.

## 4. Conclusions

The combined DM-, ITC-, and DFT-based NCI analyses demonstrate that avibactam forms thermodynamically stable 1:1 inclusion complexes with β-cyclodextrin and its C2-substituted derivatives, whereas α-cyclodextrin binds only transiently and γ-cyclodextrin exhibits markedly weaker affinity. β-CD affords the most favorable free energy of binding (ΔG° = −3.64 kcal mol^−1^; *K*_1:1_ ≈ 4.7 × 10^2^ M^−1^), while DM-β-CD and HP-β-CD benefit from stronger enthalpic contributions at the expense of higher entropic penalties, reflecting deeper guest inclusion and enhanced cavity complementarity. NCI mapping reveals that cooperative, cavity-wide van der Waals contacts—rather than the sheer number of localized hydrogen bonds—govern overall complex stability, a finding that reconciles the pronounced lability of the γ-CD system despite its multiple strong rim interactions. The congruence between calorimetric constants, MD-derived RMSD profiles, and interaction topologies provides a robust thermodynamic and structural framework that rationalizes cyclodextrin host preference for avibactam. These insights establish β-CD and its methylated or hydroxypropylated analogues as promising supramolecular platforms for supramolecular derivatization in HPLC-UV assays, for formulation strategies aimed at enhancing avibactam solubility and controlled release in critically ill patients, and for the development of cyclodextrin-based adsorbents capable of scavenging residual β-lactamase inhibitors from aqueous environments.

## Figures and Tables

**Table 1:** Standard-state binding thermodynamics of avibactam with cyclodextrins in water.

AVI/CDs	*K*_1:1_	ΔG° (kcal/mol)	ΔH° (kcal/mol)	–TΔS° (kcal/mol)
AVI/β-CD	467.29 ± 3.12	–3.64 ± 0.00	–4.57 ± 0.02	0.93 ± 0.03
AVI/DM-β-CD	236.41 ± 2.46	–3.24 ± 0.01	–7.65 ± 0.03	4.41 ± 0.04
AVI/HP-β-CD	191.57 ± 1.27	–3.11 ± 0.00	–7.08 ± 0.02	3.97 ± 0.03
AVI/γ-CD	44.25 ± 0.84	–2.25 ± 0.01	–1.42 ± 0.05	–0.83 ± 0.05

Values are means ± standard error of the fit. *K*_1:1_, 1:1 binding constant (M^−1^); ΔG°, standard Gibbs free energy of binding; ΔH°, standard enthalpy of binding; –TΔS°, standard entropic contribution.

## Data Availability

The original contributions presented in this study are included in the article/supplementary material. Further inquiries can be directed to the corresponding author.

## Supplementary Materials

The following supporting information can be downloaded at: https://www.mdpi.com/article/10.3390/molecules30163401/s1. File S1. including all input files related to docking, parameterization, and molecular dynamics simulations (topology, parameter, and configuration files), as well as optimized structures and input files for DFT and NCI analyses.
