# Supplementary figures and images for: Avibactam–Cyclodextrin Inclusion Complexes: Computational and Thermodynamic Insights for Drug Delivery, Detection, and Environmental Scavenging
Source: Molecules. 2025 Aug 18;30(16):3401. doi: 10.3390/molecules30163401 (PMC12388632; doi:10.3390/molecules30163401)

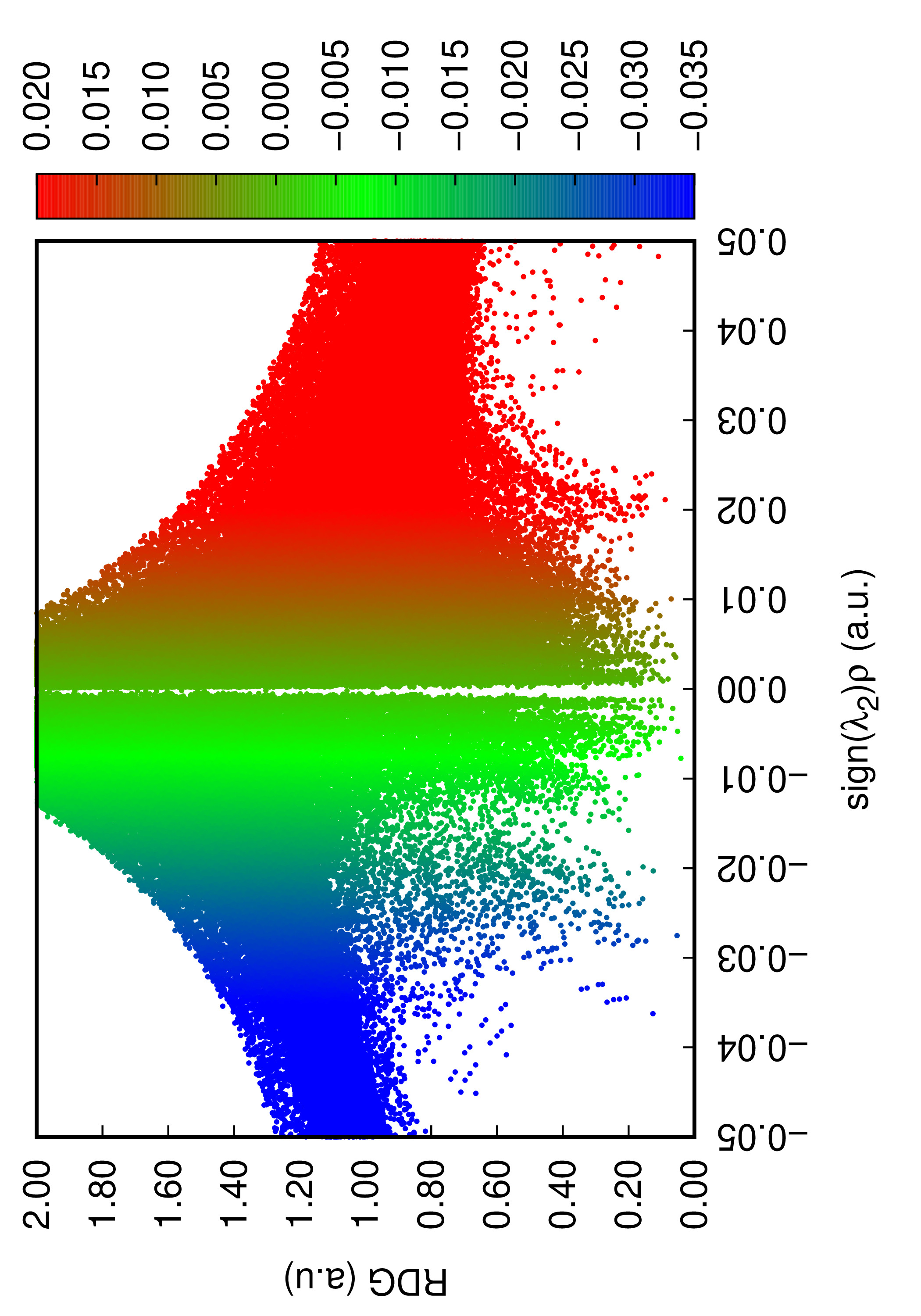

Supplement: Supplementary file 1 [file molecules-30-03401-s001.zip › DFT inputs - optimized structure - NCI/NCI/bCD/RDGscatter.jpg]

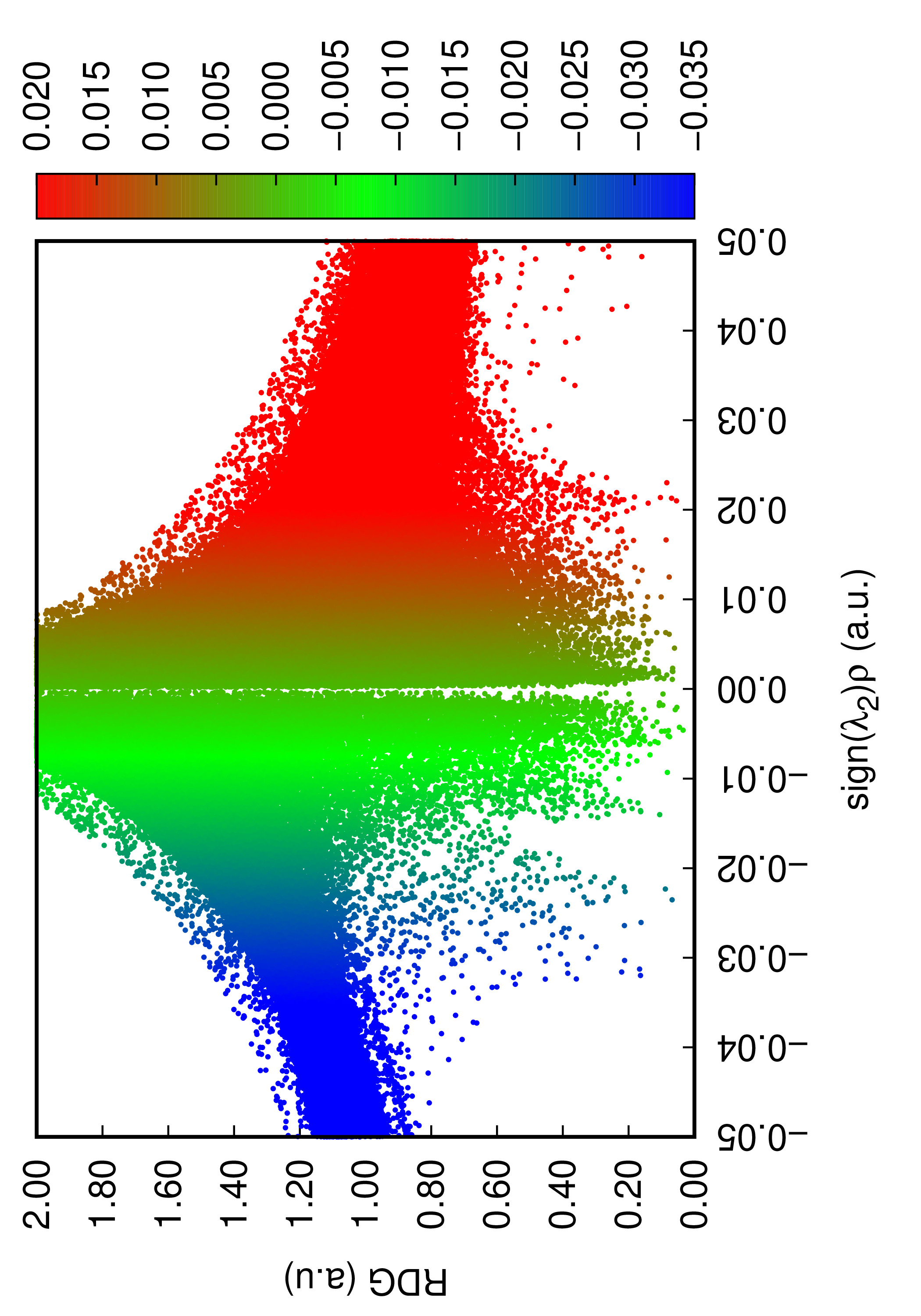

Supplement: Supplementary file 1 [file molecules-30-03401-s001.zip › DFT inputs - optimized structure - NCI/NCI/DM-bCD/RDGscatter.jpg]

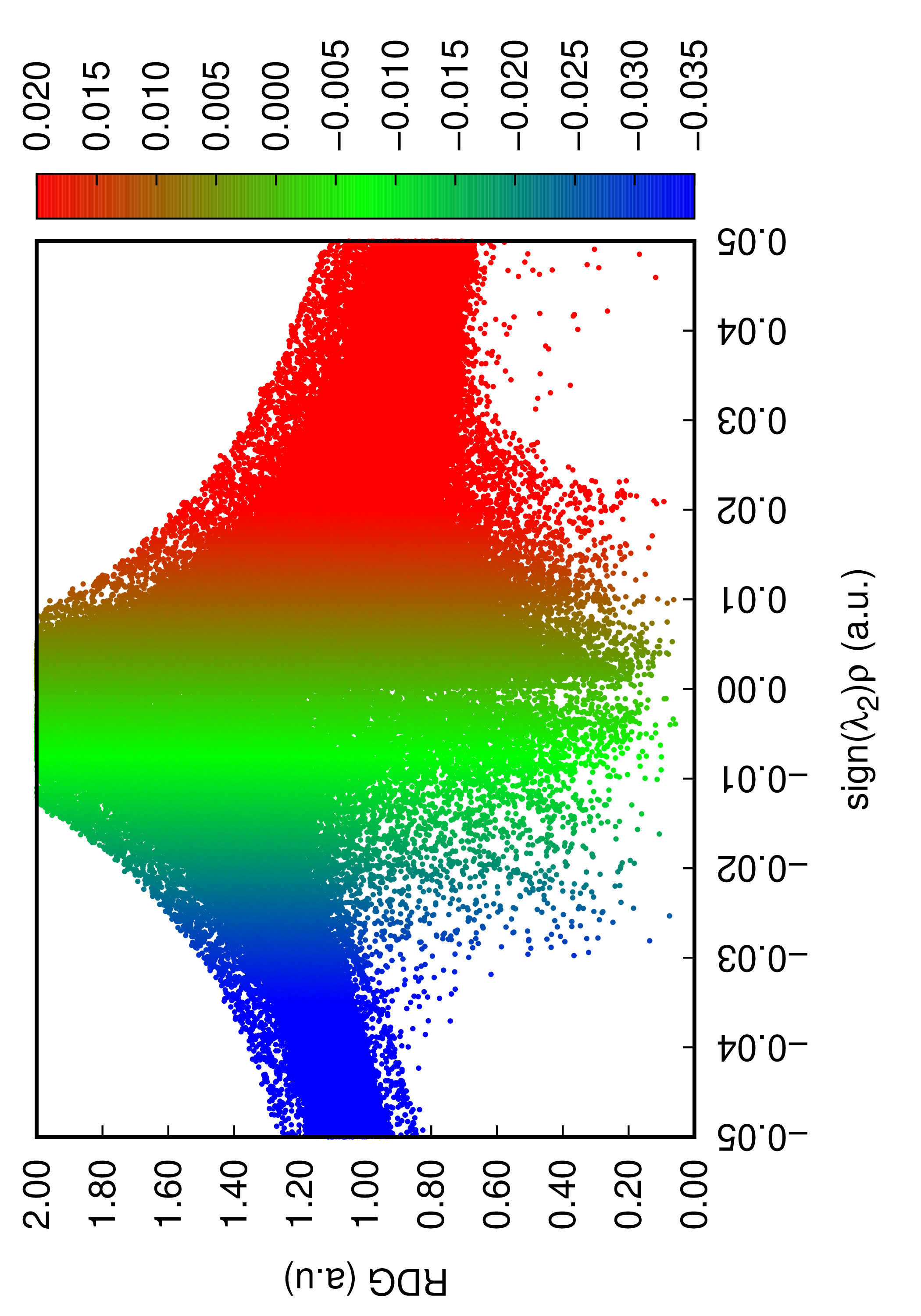

Supplement: Supplementary file 1 [file molecules-30-03401-s001.zip › DFT inputs - optimized structure - NCI/NCI/HP-bCD/RDGscatter.jpg]

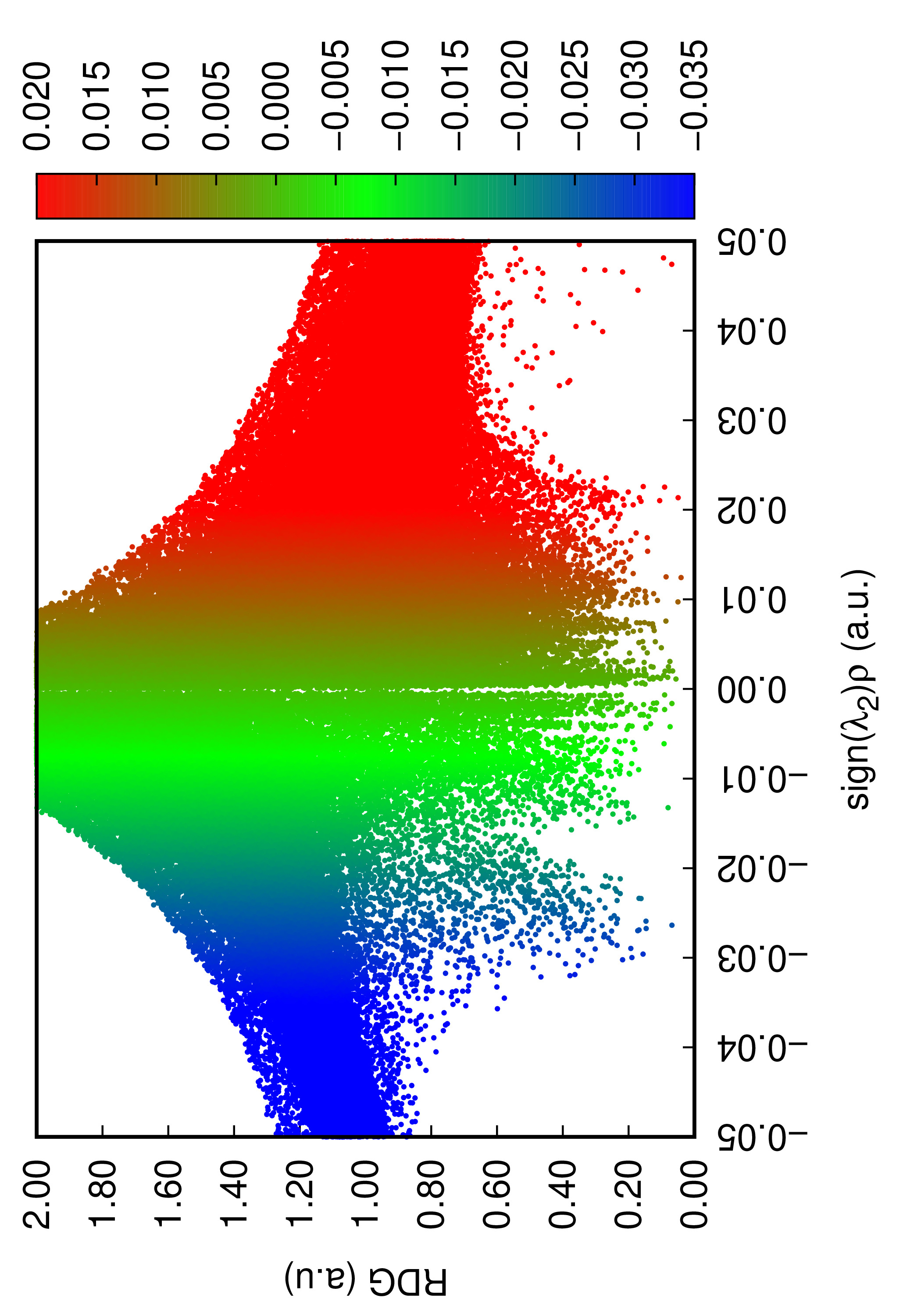

Supplement: Supplementary file 1 [file molecules-30-03401-s001.zip › DFT inputs - optimized structure - NCI/NCI/yCD/RDGscatter.jpg]
